# Electrochemical Lithium‐Ion Recovery from Battery Recycling Process Water

**DOI:** 10.1002/cssc.202502663

**Published:** 2026-04-26

**Authors:** Peter Rolf Burger, Saïd Mondahchouo, Stefanie Arnold, Moritz Goldkuhle, Sabine Flamme, Volker Presser

**Affiliations:** ^1^ INM ‐ Leibniz Institute for New Materials Saarbrücken Germany; ^2^ Department of Materials Science & Engineering Saarland University Saarbrücken Germany; ^3^ IWARU – Institute for Infrastructure Water, Resources and Environment FH Münster Münster Germany; ^4^ saarene, Saarland Center for Energy Materials and Sustainability Saarbrücken Germany

**Keywords:** battery recycling, desalination batteries, electrochemistry, lithium recovery, selective desalination

## Abstract

Electrochemical desalination is a promising technology for the selective recovery of Lithium‐ions or other rare ions from spent electronics, contributing to a circular economy. Due to its high‐energy efficiency and selective Lithium‐ion recovery, this method offers a low environmental impact, making it a promising tool for recovering Lithium‐ions from spent batteries. Few studies have examined electrochemical desalination as a tool to recover Lithium‐ions from real spent battery solutions. In this work, solutions obtained from real shredding of Lithium‐iron‐phosphate (LFP) batteries inside a cooling water reservoir were used as a Lithium‐rich source to obtain a high‐purity Lithium‐ion recovery solution. A 96%‐pure Lithium‐ion recovery solution was obtained while only requiring an energy input of 1.10 kWh/kg.

## Graphical abstract

A high‐purity Lithium‐ion solution was recovered by implementing a simple two‐step process combining the wet‐shredding of spent Lithium‐ion batteries with efficient electrochemical desalination. This work provides a simple and novel approach for the electrochemical extraction of Lithium ions from spent batteries.

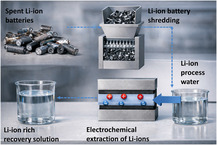

## 1 Introduction

Selective electrochemical water desalination is an essential tool for the energy‐efficient recovery of target ions [1]. This technology can be utilized as a tool to recover valuable ions, including Lithium‐ions, Neodymium‐ions, and Yttrium‐ions, as well as other rare and costly ionic species [2]. It can also be used to electrochemically remove toxic ions, such as Lead‐ions, and other undesirable ions from water [3]. Electrochemical desalination has been extensively explored in various concentrations and lab‐mixed solutions. Yet, there remains a lack of in‐depth research on its usage in real water solutions and solutions with useful practical implications [4]. Due to current and future concerns about resource depletion, finding methods to recycle products to reuse their components becomes a major requirement for the future of all technologies, especially energy storage. Lithium plays an important role as the charge carrier in most advanced electrochemical energy storage systems and thereby in the future of the automotive industry, renewable energy storage, electronics, etc. [5]. Lithium‐ion batteries have emerged as the most suitable battery choice for most modern battery‐containing technology applications due to their high‐energy and high‐power densities, and long cycling stability [6]. Even though Lithium is commonly present in nature in small quantities, there are only three sources of Lithium of significant economic importance. These consist of pegmatites, continental evaporites (such as saline lakes), and Lithium‐containing clays [7, 8]. The brine resources constitute about 78% of the total reserves [7]. Most Lithium is produced from brines in South America and Asia [8]. This geographical concentration of Lithium production can pose future risks in supply chains and thereby the energy security in non‐Lithium‐producing areas [9].

Expected future Lithium shortages in Europe and the rest of the world pose a great challenge for the next generations of humans [10]. It has been forecasted that by 2040, the material demand for Lithium and Cobalt may be exceeded by up to eight times [11]. The service life of batteries is limited and ranges from a few years to ten years, depending on battery type and load. This means a steadily growing number of used traction batteries can be expected soon [12]. Therefore, finding cost‐effective and environmentally friendly methods to recycle Lithium from spent batteries and other electronics to create a circular economy will be a significant challenge for the future. Recycling Lithium‐ion batteries enables the recovery of valuable materials, including Lithium, Cobalt, and Manganese, which can be reused in electronic products, thereby mitigating the need to extract new resources [13].

Mrozik et al. estimate that electronic waste accounts for about 4% of all landfill waste, often containing Lithium‐ion batteries [14]. Given that the rates of recycled electronics are low, most waste containing Lithium‐ion batteries is discarded into landfill sites instead of being recycled, which poses environmental risks due to the relatively high toxicity of Lithium [14]. Landfill fires and ecosystem disruption are two of many devastating impacts of Lithium‐ion batteries landing in landfills [15, 16]. These aspects all point to the significance of the circularity of Lithium‐ion batteries for the protection of our environment. Currently, most recycled products from spent Lithium‐ion batteries are recovered through pyrometallurgy and hydrometallurgy [17]. A hydrometallurgical recycling process typically begins with the full discharge of the cathode to prevent any violent reaction of the cathode once it is exposed to air [18]. The dismantling of the Lithium‐ion battery follows this. Subsequently, residual solvents and salts are removed, typically by vacuum distillation, and the binder is dissolved using a solvent, such as acetone. The electrodes are then ground and leached with an inorganic acid. Valuable metals are extracted through solvent extraction and/or precipitation, with higher value elements such as Cobalt, Manganese, and Nickel typically recovered first, before Lithium is precipitated [19]. Porvali et al. were able to recover a 95% pure Li_2_CO_3_ extract through a hydrometallurgical approach [20, 21]. However, the need for strong acids, such as HCl, and strong bases for pH adjustment poses a significant environmental burden for this method.

The pyrometallurgical approach involves the discharging of the cathode, followed by the dismantling of the cell. The next step involves the smelting of the metallic components with a reducing agent (often carbon from the graphite anode) to convert them from an oxide state to a metallic alloy [22]. In addition to the metal alloy, a slag of lighter oxides, such as Lithium oxide and Aluminum oxide, forms. Traditionally, the recovery of the minor Lithium content present in the slag has not received much attention. However, leaching and precipitation steps can be applied to recover Lithium from this slag solution. Li et al. employed a wet magnetic process to effectively separate valuable components after smelting, achieving recovery rates of 95.7%, 98.9%, and 91.1% for Co, Li_2_CO_3_, and graphite, respectively [20, 23]. Both processes require the addition of chemical reagents that produce harmful byproducts and require a relatively large amount of energy input [17]. An energy consumption of 5.82 to 2.54 kWh/kg has been reported for pyrometallurgy and hydrometallurgy, respectively [24]. Electrochemical desalination for the recovery of Lithium‐ions is an effective alternative to these methods, whereby its high Lithium‐ion selectivity of up to 50 000 for Li^+^ over other ions, low CO_2_ footprint of 9–11 g_CO2_/kWh, and low energy consumption of as little as 0.25 kWh/kg, make it an environmentally friendly method to extract high‐purity Lithium extracts [24, 25, 26, 27]. In this instance, no harmful chemicals or byproducts are created, and ions are selectively recovered from a water source. A table outlining different Lithium‐recovery technologies can be reviewed in the Supporting Information (Table S1) [28, 29, 30, 31]. Feng et al. explored LFP for 1000 cycles in an aqueous LiCl solution, and reported a 97.5% capacity retention, pointing to the potential of long‐term sustainable Lithium‐ion extraction in aqueous systems [32].

In this study, the focus is on the recovery of Lithium‐ions from a solution obtained through the industrial shredding of Lithium‐iron‐phosphate (LFP) batteries in a cooling water reservoir. A 96% pure Lithium‐ion solution was obtained while only consuming 1.10 kWh/kg energy during the entire process. Furthermore, a high average Lithium‐ion uptake capacity of 41 mg/g_LFP_ was obtained over 100 cycles.

## 2 Experimental Description

### 2.1 Structural and Chemical Characterization

The X‐ray diffraction measurements were carried out with a Bruker D8 Discover Diffractometer (Bruker AXS), with an EIGER‐2 detector, and a copper source (Cu K_α_ λ = 1.5406 Å, 40 kV, 40 mA). The powder samples were mounted onto an optical glass sample holder and analyzed from 7°‐100° 2θ with its recorded intensity normalized to (0,100).

The scanning electron micrographs were acquired using a Zeiss Gemini 500 microscope. An accelerating voltage of 2 kV was used, and the samples were mounted onto an aluminum stub using double‐sided copper tape and observed without applying an additional conductive sputter coating.

The inductively coupled plasma optical emission spectroscopy (ICP‐OES) analyses were performed using a Spectro Arcos FHX3X instrument. A nebulizer operated at a pressure of 230 kPa and a flow rate of 0.8 L/min was used to measure the specific wavelengths of each of the given elements at a plasma power of 1.3 kW. A calibration of several known samples at different concentrations was performed to relate the concentration of our ionic species to their given intensity. The intensity‐concentration equations were then used to obtain the concentration of our recovery solutions, the initial process water solution, and the diluted solid leaching solution.

Transmission electron microscopy was performed via a 2100F system (JEOL) at a voltage of 200 kV. The sample was dispersed in ethanol in an ultrasonic bath and dried onto a lacey carbon‐coated copper grid.

### 2.2 Material Leaching

For the experiments, an LFP battery cell (CALB, LFP, 3.2 V, 100 Ah, 4.35 kg) was shredded in a wet environment using a single‐shaft rotary shear shredder (Erdwich, model M700/1‐600−15, 23.5 kW) fitted with a 20 mm outlet screen. The comminution process aimed to achieve the most complete breakdown of the material possible for further processing and to separate the electrolyte via the liquid phase. As a result, numerous metal salts dissolved in the process water. As a next step, the process water was vacuum‐filtered to separate all solids and nondissolvable particles, such as graphite particles. The elemental composition of various cations in the process water was analyzed using inductively coupled plasma optical emission spectroscopy (ICP‐OES). This solution was then directly used as a Lithium‐ion‐rich solution for the recovery of Lithium‐ions.

Additionally, the Lithium content in the solid was analyzed. Thereby, the solid was dissolved in aqua regia at a temperature of 80°C and stirred for 12 h. It was then vacuum filtered to remove nondissolvable components, such as graphite. The solution obtained was then diluted and the Lithium‐ion concentration was measured via ICP‐OES. The Lithium present within the solid was not further considered for extraction and was just analyzed to gain an understanding of the overall Lithium percentage present within the liquid phase.

### 2.3 Electrode Preparation

Commercial LFP from Johnson Matthey was mixed with acetylene black conductive carbon (Alfa Aesar, 99.5%) and polyvinylidene fluoride (PVdF, Alfa Aesar) in 1‐methyl‐2‐pyrrolidone (NMP, Sigma–Aldrich, 99.5%) in a mass ratio of 8:1:1. The powder components were combined and mixed at 1000 rpm for 5 min using a Hauschild SpeedMixer DAC 150 SP. To achieve the desired slurry consistency, NMP was added gradually while mixing. The mixture was then processed sequentially at 1500 rpm for 5 min and 2500 rpm for another 5 min. A 10 mass% solution of PVdF binder in NMP was incorporated last, and the final electrode paste was mixed at 800 rpm for 10 min. Homogenization of the slurry was achieved by magnetic stirring for 12 h. Subsequently, the slurry was deposited onto graphite paper (SGL, 300 μm substrate) via doctor‐blade coating, producing a wet film thickness of 200 μm. After drying overnight at room temperature, the films were vacuum‐dried at 100°C for 12 h to evaporate residual solvent. Disks of 30 mm diameter were punched from the dried coating for use as working electrodes. The dried electrode coating had a thickness of 65–70 μm and a material loading of 3.9 mg/cm^2^.

### 2.4 Electrochemical Cell Assembly and Experimental Details

Two types of electrochemical desalination cells were assembled and used to extract Lithium. Cell 1 was cycled in a feed solution containing an aqueous lab‐mixed solution with the same concentrations of cations as found in the process water, bonded to chloride anions. Cell 2 was cycled in the actual shredded battery process solution. From here on, Cell 1 and Cell 2 are in reference to the electrolyte in which they are cycled. During the discharging steps of the electrochemical desalination experiments, a separate 100 mL recovery solution, initially with a concentration of 10 mM KCl, was used to discharge the stored Lithium‐ions into.

For the electrochemical characterization tests, custom‐built static electrochemical cells were assembled in a three‐electrode setup. These are comprised of a polyether ether ketone (PEEK) outer body with spring‐loaded titanium pistons, a carbon‐coated LFP electrode, and an oversized commercial activated carbon cloth (Kynol‐15−5092) was used as the counter electrode (CE) [33]. Two Glass fiber separators (GF/A, 210 μm thickness, Whatman) were employed to prevent short‐circuiting between the two electrodes, and these components were arranged in a stacked configuration [34]. In a hole close to the working and CE, an Ag/AgCl reference electrode is positioned. The electrolyte was inserted into the cell by vacuum‐backfilling. Figure S3A shows a photograph of an assembled static electrochemical cell.

The Lithium‐ion recovery experiments were conducted in a custom flow electrochemical cell consisting of an acrylic glass channel that allows for the continuous flow of solution through the electrochemical cell [35]. Silicon gaskets are employed throughout the cells to avoid leakage; graphite blocks were used as the current collectors of the cell. Both the working and CEs were cut into 30 mm circular electrodes and were placed into contact with the graphite current collectors. The electrodes are separated via the acrylic flow channel. The interior of the flow channel consists of a circular hole with a 28 mm diameter that is filled with nylon mesh and glass fiber separators to avoid short‐circuiting and to allow for good contact between the current collectors and the electrodes. Figure S3B shows a photograph of an assembled flow electrochemical cell.

To allow for the continuous operation of the system, an anion exchange membrane (Fumatech, FAS‐30) was employed for Cell 1 [36]. This prevented the undesirable expulsion of cations (co‐ions) from the CE during discharge, thus ensuring that the CE's activity did not influence the recovery solution's cation concentration. Due to the nature of the shredded battery solution, organic components and larger particle impurities led to the clogging of the anion exchange membrane (AEM) in Cell 2, which was subsequently cycled without an AEM. To avoid the issues associated with co‐ion expulsion from the CE during discharge, the cell was carefully washed with ultrapure water, followed by the replacement of the CE with a new one before each discharging step.

The concentration changes of Li^+^, Na^+^, Mg^2+^, Mn^3+^, and Co^3+^ in the recovery solution were measured using ICP‐OES [37], and these changes in concentration were used to determine the cation composition and thereby the Lithium purity. The average number of recovered ions per cycle was taken to determine the Lithium purity and the desalination capacity of the cell. In this way, the Lithium‐ion purity was determined by dividing the number of recovered Lithium ions by the total number of recovered ions.

## 3 Results and Discussion

Electrochemical desalination enables the removal of dissolved ions from a water stream by applying electrical current across an electrochemical cell. The polarization due to the induced electrical potential attracts the ions in the water to the oppositely charged electrode, resulting in their electrochemical uptake [38]. During charging, the cations migrate toward the negatively polarized electrode, while the anions migrate toward the positively polarized electrode [27]. These ions are then released back into a separate solution during discharging, allowing for their recovery. Electrochemical desalination can be used to selectively extract Lithium‐ions from a solution when a reversible Lithium‐ion‐selective electrode material or membrane is employed. The cell can be operated at ambient temperatures, without the input of toxic chemicals, and it does not require additional energy input besides the electricity that drives the Lithium uptake and the pump that continuously circulates the solution through the cell. A feed solution, derived through the shredding of LFP batteries inside a cooling water reservoir, supplies the Lithium‐ions during the charging step of the electrochemical desalination. The Lithium ions are intercalated into the LFP structure during charging. During the discharging step, these Lithium‐ions are recovered from the LFP as they deintercalate from the LFP structure. The Lithium‐ions are discharged into a separate aqueous recovery solution. LFP was used as working electrode material due to its high selectivity for Li^+^ over other competing ions, such as Na^+^ or Mg^2+^. This selectivity is primarily governed by two phenomena: size and kinetics, and charge and hydration. Size and kinetics: LFP possesses an olivine crystal structure with one‐dimensional channels along the [010] direction. While Li^+^ (∼0.76 Å) can move freely through these channels, larger ions, such as Na^+^ (∼1.02 Å), have a kinetic bottleneck where they are excluded from entering the lattice due to their size. The intercalation of Na^+^ occurs based on cyclic voltammetry data at a higher overpotential, whereby bulk intercalation of Na^+^ can be avoided by excluding the potential window where bulk Na^+^ ion intercalation occurs [39].

Charge and hydration: Even though Mg^2+^ (0.72 Å) has an ionic radius nearly identical to Li^+^, its divalent charge creates a much stronger electrostatic interaction with the oxygen atoms in the phosphate lattice, making it energetically unfavorable to be inserted into these sites. In addition, its higher hydration energy is a massive barrier for its insertion into the lattice, as it requires a larger energy to strip the water molecules off the Mg^2+^ [25].

**FIGURE 1:**
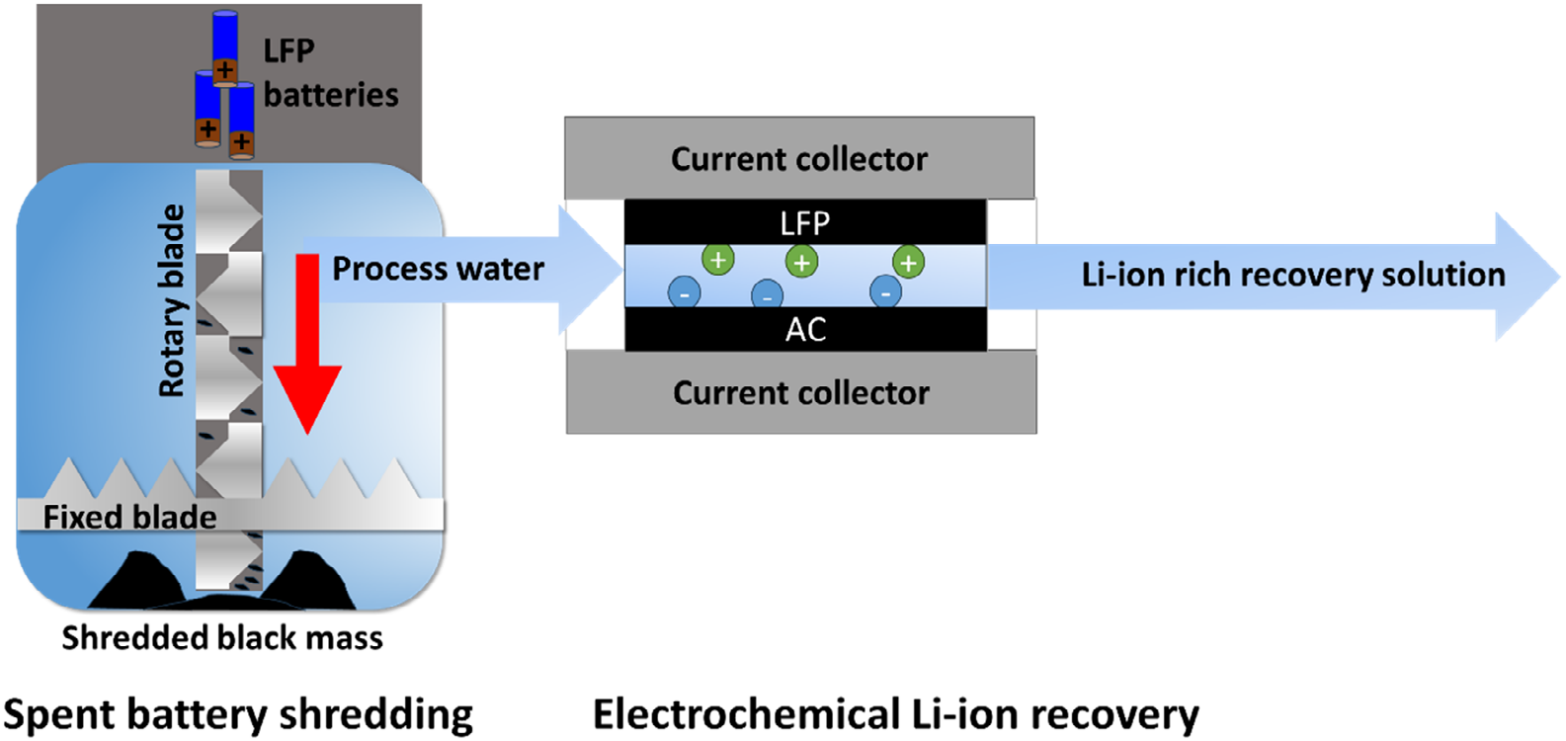
LFP batteries are shredded under process water, producing a Lithium‐rich aqueous stream. This stream is pumped through an electrochemical cell, where an applied potential selectively captures Lithium‐ions from solution. During charging, the process water is desalinated as Lithium‐ions are removed; during discharging, the captured Lithium is released into a separate recovery solution.

Figure 1 shows a simplified setup of how Lithium ions are recovered from batteries. LFP batteries were shredded inside a process water reservoir for 2.5 min. This solution was then filtered and pumped through an electrochemical cell. An electrical current is applied across the cell, resulting in the uptake of Lithium‐ions. These ions are then released into a separate solution (recovery solution) from the cell during discharge. Over multiple cycles, the Lithium‐ion concentration of the recovery solution increases, resulting in a Lithium‐ion‐rich recovery solution. The Lithium‐ion‐rich solution can then be further processed into a solid Lithium salt through further processing steps, such as evaporation. The composition of this solid salt is dependent on the anion composition and can include Li_2_CO_3_, LiCl, or other Lithium‐ion‐containing salts.

Furthermore, some of the energy invested during the charging step can be recovered during the discharging step and used to power additional desalination cells or other electronic equipment. The system can then be considered a desalination battery, whereby Lithium‐ions can be recovered and energy can be stored electrochemically [40]. Important considerations regarding the other rare elements (Cobalt, Nickel, etc.) present in Lithium‐ion batteries cannot be neglected either, and additional steps to recover those might require some leaching and/or temperature treatment that is not discussed in this paper.

As the first step of the procedures, the LFP batteries were shredded, and the obtained liquid solution was analyzed via ICP‐OES. Upon analyzing the elemental composition of various cations in the electrolyte using ICP‐OES, the concentrations as presented in Table 1 were obtained. 12 different elements were measured, assuming that these elements, aside from Lithium, may be present within the solution. A concentration of 45.76 mM of Lithium‐ ions was detected, while the concentration of all the other competing ions detected was relatively low. The second largest ionic concentration found was that of Sodium‐ions with 0.71 mM. Other elements at varying concentrations may be present in the solution; however, to our best knowledge, these elements were the most likely competing ions dissolved in the shredded battery solution. For all the other measured cations (Al^3+^, Ca^2+^, Co^2+/3+^, Fe^3+^, K^+^, Mg^2+^, Mn^2+/4+/7+^, Ni^2+^, Zn^2+^, Cu^+/2+^), a concentration of less than 0.5 mM was found in the process water solution. Qin et al. reported a nearly identical Lithium‐ion extraction rate and ion selectivity for LiCl solutions ranging from 0.1 to 1 M concentration [41]. Thereby, ensuring that the concentration of the liquid is sufficient for optimal extraction rates.

**TABLE 1:** Cation concentrations in the shredded battery solution. n.d.* = not detected.

	Concentration, mM
Aluminum	0.41
Calcium	0.18
Cobalt	0.18
Iron	0.10
Potassium	0.13
Lithium	45.76
Magnesium	0.19
Manganese	0.31
Sodium	0.71
Nickel	0.05
Zinc	0.00
Copper	n.d.*

In addition, the solid product that was filtered out was analyzed via ICP‐OES to confirm the amount of Lithium present within it. The solid was leached in aqua regia for 12 h to ensure the full dissolution of all Lithium ions present within the solid product. Based on the solid‐to‐liquid ratio within the cooling water reservoir (3.5 g of solid material per 1 L of process water solution), the total percentage of Lithium‐ions in the solid was determined to be 12.3%, and the Lithium content in the liquid was 87.7%.

This Lithium‐ion‐rich process solution was then used as a feed water source for the remaining electrochemical desalination tests. The Lithium content present in the solid was not further considered for these Lithium‐ion recovery studies.

The LFP electrode was characterized by scanning electron miscorscopy (SEM), transmission electron microscopy (TEM), and X‐ray diffraction (XRD). The scanninge electron micrographs of pristine and postmortem samples show a small particle size of 10–100 nm and the postmortem electrode shows no noticeable difference in morphology after cycling for 100 cycles to the pristine material (Supporting Information, Figure S1). The primary darker particles in the transmission electron micrograph can be associated with the olivine LFP structure. A lighter, less‐defined matrix surrounding these LFP particles aligns with the presence of an amorphous carbon coating (Supporting Information, Figure S2B). XRD analysis confirms the olivine phase and high crystallinity of the pristine material (Supporting Information, Figure S2A). The XRD analysis of the post mortem material, after cycling the cell for 100 cycles in the process water solution, shows a significant decrease in the 2θ position of the (200), (311), and (020) reflections, indicating a lattice expansion of the crystal lattice along these planes due to repeated Lithium‐ion intercalation.

**FIGURE 2:**
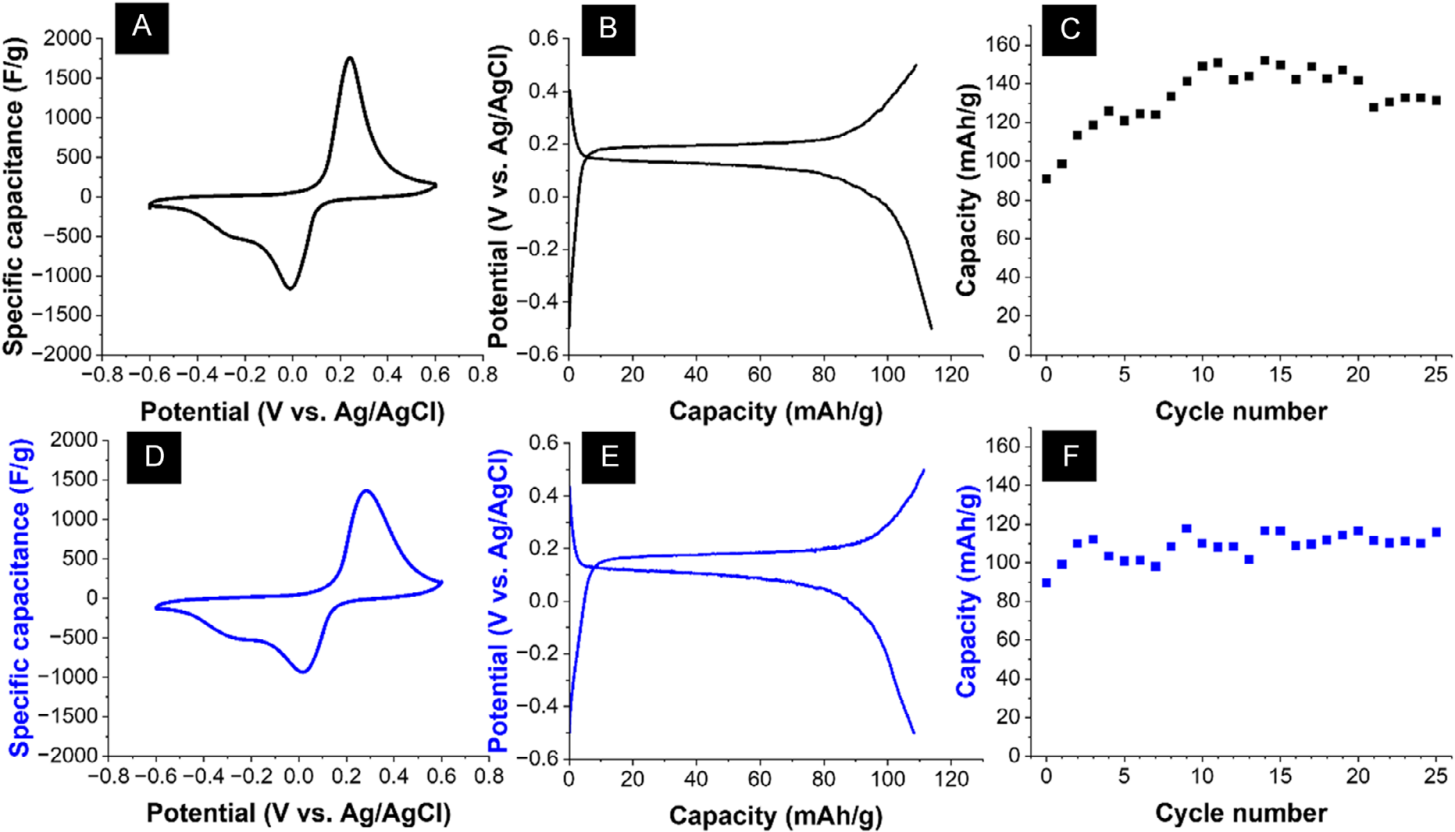
(A) Cyclic voltammogram of the LFP electrode performed at a scan rate of 1 mV/s in the lab‐mixed electrolyte in a static cell setup. (B) Galvanostatic charge and discharge profiles at a specific current of 0.05 A/g were performed in the lab‐mixed electrolyte. (C) Galvanostatic charge‐discharge cycling performance at a specific current of 0.05 A/g at a charging potential of ‐0.5 V vs. Ag/AgCl and a discharging potential of +0.5 V vs. Ag/AgCl was performed in the lab‐mixed electrolyte. (D) Cyclic voltammogram of the LFP electrode performed at a scan rate of 1 mV/s in the process water solution in a static cell setup. (E) Galvanostatic charge and discharge profiles at a specific current of 0.05 A/g in the process water solution. (F) Galvanostatic charge‐discharge cycling performance at a specific current of 0.05 A/g at a charging potential of ‐0.5 V vs. Ag/AgCl and a discharging potential of +0.5 V vs. Ag/AgCl performed in the process water solution.

A static electrochemical cell consisting of an LFP working electrode was tested both in the lab‐mixed electrolyte and in the shredded process solution. The system was electrochemically characterized by cyclic voltammetry performed at a scan rate of 1 mV/s, as shown in Figure 2A,D for the cell cycled in the lab‐mixed electrolyte and for the cell in the process water solution, respectively. The pronounced characteristic redox peaks, as shown in both voltammograms, can be associated with the intercalation of Lithium ions into and out of the structure of the LFP. In addition, both cells show a slight reduction peak at approximately −0.25 V vs. Ag/AgCl, which could be associated with the intercalation of another ionic species into the LFP structure, possibly Sodium ions. However, this could not be confirmed, and further studies must be conducted in different electrolytes to confirm this.

Galvanostatic charge–discharge cycling was performed in both electrolytes for the static cell setup at a specific current of 0.05 A/g and a charging potential of −0.5 V vs. Ag/AgCl and a discharging potential of 0.5 V vs. Ag/AgCl. Here, Figure 2B,C shows the charge and discharge profiles of the cell in the lab‐mixed electrolyte and its capacity for the first 25 cycles. The cell has a high average capacity of 133 mAh/g. Figure 2E,F shows the charge and discharge profiles of the cell in the real battery process water solution and its capacity for the first 25 cycles. The cell has an average capacity of 109 mAh/g. Both the electrochemical signature and the average capacity point show nearly identical behavior between the two cells. Arnold et al. recorded an initial capacity of 123 mAh/g when cycling carbon‐coated LFP in a LiCl solution, which drops off to approximately 100 mAh/g after 20 cycles, showing that both static electrochemical cells exhibit a comparable capacity to similar studies [42].

As a next step, flow electrochemical cells were tested in both the lab‐mixed electrolyte and the process water solution to test for their Lithium‐ion recovery capacities. Before the first charging step, a cell potential of 1.0 V was applied to deintercalate the Li‐ions present in the structure of the LFP electrode. To recover the Lithium‐ions from the solutions, after a washing step with deionized water, electrochemical charging was conducted at a charging cell potential of −0.5 V and a specific current of 0.05 A/g. The ions were discharged into a recovery solution, initially consisting of 10 mM of KCl, at a cell potential of 0.5 V and a specific current of 0.05 A/g.

**FIGURE 3:**
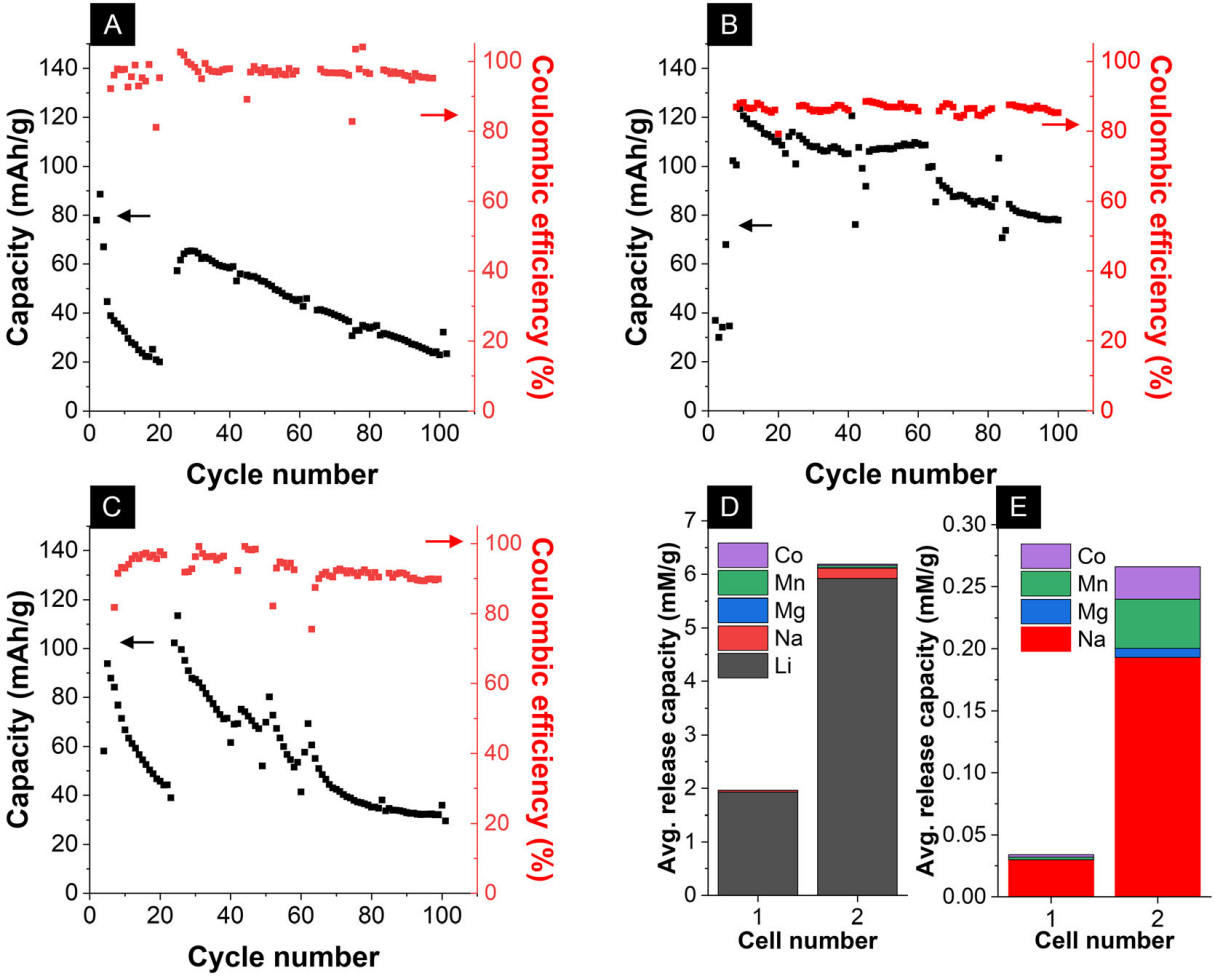
Electrochemical data obtained by the cycling of the flow electrochemical cell. (A), (B), (C) Capacities and Coulombic efficiencies of Cell 1, Cell 2, and Cell 3, respectively, over 100 cycles in the flow cell setup. (D) Total average release capacity accounting for all its individual ionic constituents. (E) Shows the total average release capacity for all ionic species excluding Lithium‐ions.

The electrochemical performance of the Lithium‐ion recovery cells is compared across 100 cycles using the lab‐mixed electrolyte (Cell 1, Figure 3A) and the process water solution (Cell 2, Figure 3B). Cell 1 exhibits a high initial capacity, followed by a sharp drop in capacity, before recovering to a peak capacity of around 65 mAh/g. This anomaly is likely due to the irreversible consumption of a temporary surface phase, followed by a period where the bulk LFP is still undergoing activation before reaching its full potential. However, its capacity stability is poor, dropping steeply to less than 25 mAh/g after 100 cycles. Cell 2 shows a more typical behavior, starting at a low capacity and rapidly rising to its peak of 120 mAh/g after 10 cycles. This initial low capacity points to a high internal resistance, likely caused by the incomplete wetting of the LFP particles. As cycling proceeds, the electrode/electrolyte interface stabilizes and the particles become fully wetted, allowing access to the bulk material and achieving a larger sustained capacity (80 mAh/g after 100 cycles).

A third electrochemical flow cell (Cell 3) was assembled and cycled in the lab‐mixed solution without an AEM, and experiments were carried out identically to those for Cells 1 and 2. Figure 3C shows the electrochemical performance for Cell 3 over 100 cycles. The cell has a nearly identical behavior to Cell 1, whereby it initially exhibits a high initial capacity, followed by a sharp drop in capacity, before recovering to a peak capacity of around 113 mAh/g. This anomaly is likely due to the irreversible consumption of a temporary surface phase, followed by a period where the bulk LFP is still undergoing activation before reaching its full potential. However, its capacity stability is poor, dropping steeply to 30 mAh/g after 100 cycles. It has a higher average capacity than Cell 1 but a significantly lower capacity than Cell 2. The discrepancy between the cells cycled in the artificial solution vs. the process water solution cannot be solely understood by the AEM's presence in the cell and is likely due to some irreversible consumption of the LFP in the artificial battery solution.

To evaluate the long‐term performance for Lithium‐ion extraction, the ion concentration of the recovery solution was analyzed after a total of 15 discharge cycles, distributed across the 100‐cycle stability test. The final recovery solution concentration was measured for both cells 1 and 2. Based on the total concentration averaged over 15 discharge cycles, Cell 1 has an average cyclic release capacity of 13 mg/g or 1.9 mM/g_LFP_. Cell 2 has a significantly higher average Lithium‐ion release capacity of 41 mg/g or 5.9 mM/g_LFP_. The recovery capacity of Cell 2 is close to the theoretical limit of LFP (44 mg/g). This likely stems from some residual salts adhering to cell components or tubing, which were inadvertently included in the concentration analysis despite the washing procedure. Not all the Lithium ions present in the recovery solution can be attributed to electrochemical activity and mechanical entrainment (brine carry‐over) within the porous architecture of the electrode, which must account for some of the ions present within the solution as well. This entrainment could be due to the ineffectiveness of the rinsing protocol and thereby result in slightly inflated values in the ion concentrations. However, the large discrepancy between the two cells’ Lithium‐ion recovery is directly reflected by the two cells’ large capacity difference over 100 cycles, showcasing that electrochemical recovery is the primary mechanism associated with the Lithium‐ion recovery in Cell 2.

Figure 3D shows the complete cation composition of the recovery solution. For Cell 1, the second largest recovery capacity is for Sodium‐ions with a recovery capacity of 0.6 mg/g or 0.03 mM/g_LFP_. For Cell 2, the second largest recovery capacity is also for Sodium‐ions with a recovery capacity of 4.0 mg/g or 0.2 mM/g_LFP_. The average recovery capacity for the remaining ions (Mn‐ions, Mg‐ions, and Co‐ions) is less than 0.05 mM for both cells. Figure 3E shows the average ionic composition of the recovery solution for all the elements besides Lithium‐ions to allow for an easier representation of the other average ionic concentrations. Cell 1 exhibited a 98% pure Lithium‐ion recovery solution, while Cell 2 exhibited a 96% pure Lithium‐ion recovery solution. The remaining cations present in the recovery solution (impurity cations) are primarily comprised of Sodium ions for both cells 1 and 2.

The total average desalination capacity over 100 cycles, considering all measured ions, was 14 mg/g for Cell 1 and 49 mg/g for Cell 2. The large discrepancy between these two values is likely due to the irreversible consumption associated with Cell 1 or also due to Cell 1's AEM that causes a larger kinetic barrier, resulting in a longer time for the full activation of the LFP particles. The desalination capacity achieved for the battery solution (Cell 2) is higher compared to most reported literature. For instance, the system developed by Xu et al. demonstrated a lower desalination capacity of 27 mg/g in a brine solution. In contrast, our system achieves a capacity of 49 mg/g, which shows the promising performance of the system in the process water solution [43].

An energy expenditure of 1.10 kWh/kg is expected for Cell 2, based on the analysis of the total energy required to extract Lithium‐ions. Here, this value encompasses the energy consumption of the shredding process (0.5 kWh/kg), the pumping process (0.1 kWh/kg), and the electricity required to charge the cell (0.5 kWh/kg). The calculations used to determine energy consumption and the performance metrics of the electrochemical cell are described in detail in the Supporting Information document.

Based on energy expenditure, it has a lower energy consumption than both pyrometallurgical and hydrometallurgical processes by 4.72 and 1.44 kWh/kg, respectively. This electrochemical Lithium‐ion extraction method eliminates the preroast step that is required for hydrometallurgical and pyrometallurgical processes, making it significantly less energy intensive. Additionally, it does not require the use of toxic chemicals or create any toxic byproducts during the process. This makes it a promising and greener alternative to effectively extract Lithium ions from spent battery solutions.

## 4 Conclusions

The electrochemical cell, consisting of an LFP working electrode, exhibits high selectivity toward Lithium‐ions, thereby producing a high‐purity Lithium‐ion recovery solution through electrochemical extraction. The Lithium‐ion‐rich recovery solutions obtained from cells 1 and 2 have purities of 98% and 96%, respectively. Cell 2's high Lithium‐ion recovery capacity of 41 mg/g and low energy consumption of 1.10 kWh/kg make it an environmentally friendly alternative to other Lithium‐ion recycling methods, without any toxic byproducts. Further processing steps could be conducted to extract other valuable resources from the solid products acquired after shredding the batteries. After the filtration of the high‐concentration Lithium‐ion solution, the obtained black mass could be processed via leaching or smelting to obtain other rare battery constituents.

Additionally, further studies are needed to confirm whether the shredding process for other Lithium‐ion battery chemistries (LMO, NMC, LCO, etc.) yields an equally suitable solution for the electrochemical recovery of Lithium ions. Despite its high performance, the manual labor of changing the CE after the cycling does not allow for continuous operation due to the clogging of the AEM. Further studies are needed to develop a system that integrates a working AEM unaffected by the organic components present in the electrolyte.

## Supporting Information

Additional supporting information can be found online in the Supporting Information section. It contains details on the energy expenditure calculations for each process step associated with the recovery of Lithium‐ions. In addition, it contains details on the formulas used for the calculations of the electrochemical desalination performance metrics (desalination capacities, Lithium purity, etc.). It also contains figures characterizing the LFP electrode.

## Author Contributions

**Peter Burger**: investigation, conceptualization, data curation, visualization, writing – original draft, writing – review & editing. **Saïd Mondahchouo**: investigation, writing – review & editing. **Stefanie Arnold**: investigation, writing – review & editing. **Moritz Goldkuhle**: investigation, writing – review & editing. **Sabine Flamme**: writing – review & editing. **Volker Presser**: conceptualization, supervision, validation, resources, visualization, writing – original draft, writing – review & editing, project administration, funding acquisition.

## Funding

This study was supported by Deutsche Forschungsgemeinschaft (PR 1173/33), European Union from the European Regional Development Fund (eLiFlow), State of Saarland (EnFoSaar).

## Conflicts of Interest

The authors declare no conflicts of interest.

## Lead Contact

Requests for further information, resources, or materials should be directed to and will be fulfilled by the lead contact upon request, Volker Presser (volker.presser@leibniz-inm.de).

## Materials Availability

The authors have nothing to report.

## Data and Code Availability

The data generated in this study are included in the manuscript and Supporting information and will be made available from the lead contact upon request.

## Supporting information

Supplementary Material

## Data Availability

The data that support the findings of this study are available via zenodo https://doi.org/10.5281/zenodo.19681336.
