# Supplementary material for: Electrochemical Lithium‐Ion Recovery from Battery Recycling Process Water
Source: ChemSusChem. 2026 Apr 26;19(8):e202502663. doi: 10.1002/cssc.202502663 (PMC13110910; doi:10.1002/cssc.202502663)
Supplement: Supplementary file 1 — Supplementary Material [file CSSC-19-e202502663-s001.pdf]

Supporting Information

Electrochemical Lithium-ion recovery from battery recycling process water

Peter Rolf Burger,[1,2] Saïd Mondahchouo,[1,2]

Stefanie Arnold,[2] Moritz Goldkuhle,[3] Sabine Flamme[3]

and Volker Presser[1,2,4,]*

1 *INM - Leibniz Institute for New Materials, D2 2, 66123, Saarbrücken, Germany*

2 *Department of Materials Science & Engineering, Saarland University, Campus D2 2, 66123, Saarbrücken, Germany*

3 *IWARU – Institute for Infrastructure, Water, Resources and Environment, FH Münster, Correnssstraße 25, 48149 Münster, Germany*

4 *saarene, Saarland Center for Energy Materials and Sustainability, Campus C4 2, 66123 Saarbrücken, Germany*

* Corresponding author's email: volker.presser@leibniz-inm.de

Calculations

Calculation of ion recovery based on ICP-OES results:

For each analyzed cycle, an ICP-OES measurement of Li^+, Na^+, Mg^{2+}, Co^{3+}, and Mn^{3+} was taken.

The number of recovered ions (RI) of a given species that was recovered was calculated by **Eq. (1)**:

$$RI\ (mmol) = (C_{ad} * V_{ad}) - (C_{bd} * V_{bd}) \quad (1)$$

where C_{ad} represents the concentration of a given ion within the recovery solution after discharge, V_{ad} represents the volume of the recovery solution after discharge, C_{bd} represents the concentration of a given ion within the solution before discharge, V_{bd} represents the volume of the recovery solution before discharge.

The RI was then in turn used to calculate the recovery capacity (RC) of each of the ionic species per gram of electrode material (M_{LFP}) by **Eq. (2)**:

$$RC\ \left(\frac{mM}{g}\right) = RI\ /\ M_{LFP} \quad (2)$$

This could then in turn be multiplied by the molar mass of a given ionic species to obtain the RC in mg/g. The Lithium-ion recovery capacities as plotted in **Figure 3D** were calculated according to these calculation steps.

Calculation of the purity of the recovery solution:

The purity of the Lithium-ion recovery solution was calculated by **Eq. (3)**:

$$Li^+\ Purity\ (\%) = \frac{\sum_{j=1}^{M} N_{Li,j}}{\sum_{j=1}^{M} N_{all,j}} \times 100 \quad (3)$$

where $N_{Li,j}$ represents the number of Li^+ ions recovered in measurement j, and $N_{all,j}$ represents the total number of recovered ions of all species in measurement j. M represents the total number of measurements performed.

Energy expenditure calculations:

For the energy expenditure 3 aspects were considered:

1) The shredding process
2) The energy input during the charging/discharging of the electrochemical cell
3) The pumping process

1) For the shredding process, the energy expenditure was calculated as follows:

   For one LIB cell of 4.35 kg, 2 min 26 sec of shredding was considered. With a drive power of 23.5 kW this resulted in total energy consumption of 0.95 kWh or 0.22 kWh/kg. This was then normalized to the mass of the LFP electrode for the ease of comparison with other literature. Hereby, it is assumed that LFP constitutes 43 % of the total battery module. Thereby, an energy expenditure of $0.51\ kWh/kg_{LFP}$ is assumed for the shredding process.

2) An average energy consumption of 0.02 Wh/g during the charging step was taken, and an average Lithium-ion recovery capacity of 41 mg/g was used to calculate the energy consumption associated with the charging of the cell. The energy consumption associated with the cycling of the cell was calculated by **Eq. (4)**:

$$E_{electric} = \frac{E_{charge}}{RC} = \frac{0.02\frac{Wh}{g_{LFP}}}{0.041\frac{g_{Li+}}{g_{LFP}}} = 0.49\frac{kWh}{kg_{Li+}} \quad (4)$$

   This value of $0.49\frac{kWh}{kg_{Li+}}$ is likely higher than what the "real energy consumption" would be as during discharge some of the energy could be recovered, which is not accounted for in this calculation.

3) For the pumping process, 4 h of continuous pumping was considered, which is the approximate average pumping time per cycle. This was multiplied by the power of the pump, which was determined for its specified operational parameters (flow rate = 5 mL/min, tube diameter = 0.22 cm, tube length = 50 cm, and the fluid was treated as water regarding viscosity and density). The power of the pump was calculated via **Eq. (5)**:

$$P_{pump} = \frac{p \times Q}{3.6 \times 10^6 \times \eta} \tag{5}$$

Where p is the hydraulic pressure (Pa), Q is the flow rate of the solution $(\frac{m^3}{h})$, and η is the pump efficiency. The pump efficiency is estimated to be 30 % (assuming a low efficiency at low flow rates as the ones used in this experimental setup). The hydraulic pressure was calculated by **Eq. (6)**:

$$p = \rho \times g \times h \tag{6}$$

where ρ is the density of the fluid (kg/m^3), g is gravity (m/s^2), and h is the total head across the pump (m).

The total head loss was calculated via **Eq. (7)**:

$$h = (h_d - h_s) + h_f + \frac{V_d^2 - V_s^2}{2g} \tag{7}$$

where the velocity head $\left(\frac{V_d^2 - V_s^2}{2g}\right)$ is neglected as one assumes the velocity of suction and velocity of discharge are equal. In addition, the static head $h_d - h_s$ (vertical distance between suction and discharge points) is also neglected as the liquid is suctioned and discharged to/from the same liquid source. h_f (friction losses) are calculated using the Darcy-Weisbach equation **Eq. (8)**:

$$h_f = f \times \frac{L}{D} \times \frac{V^2}{2g} \tag{8}$$

where f is Darcy friction factor (dimensionless), L is the length of the pipe (m), D is the inner diameter of the pipe (m), V is the average velocity of the fluid $\left(\frac{m}{s}\right)$, and g is gravity $\left(\frac{m}{s^2}\right)$.

Supporting Tables

Table S1: Comparison of key performance indicators of major Lithium extraction technologies, comparing a Pyrometallurgical process, a Hydrometallurgical process, a direct process, an electrolysis process, an electrochemical process, and this work's electrochemical process.

Li-extraction method	Total energy consumption (kWh/kg)	Lithium purity (%)
**Pyrometallurgy[1]**	5.82	> 99
**Hydrometallurgy[2]**	2.54	96
**Direct[3]**	7.76	99.5
**Electrolysis[4]**	7.64	99
**Electrochemical[5]**	0.25	99
**This work**	1.10	96

Supporting References

1. Xiao, J., Li, J., and Xu, Z. (2017). Recycling metals from lithium ion battery by mechanical separation and vacuum metallurgy. Journal of hazardous materials *338*, 124-131.
2. Li, H., Xing, S., Liu, Y., Li, F., Guo, H., and Kuang, G. (2017). Recovery of Lithium, Iron, and Phosphorus from Spent LiFePO4 Batteries Using Stoichiometric Sulfuric Acid Leaching System. ACS Sustainable Chemistry & Engineering *5*, 8017-8024. 10.1021/acssuschemeng.7b01594.
3. Yang, L., Gao, Z., Liu, T., Huang, M., Liu, G., Feng, Y., Shao, P., and Luo, X. (2023). Direct electrochemical leaching method for high-purity lithium recovery from spent lithium batteries. Environmental science & technology *57*, 4591-4597.
4. Li, Z., He, L., Zhu, Y., and Yang, C. (2020). A green and cost-effective method for production of LiOH from spent LiFePO4. ACS Sustainable Chemistry & Engineering *8*, 15915-15926.
5. Wang, W., Liu, Z., Zhu, Z., Ma, Y., Zhang, K., Meng, Y., Ahmad, T., Khan, N.A., Peng, Q., and Xie, Z. (2025). Electrochemical lithium recycling from spent batteries with electricity generation. Nature Sustainability *8*, 287-296.

Supporting Figures

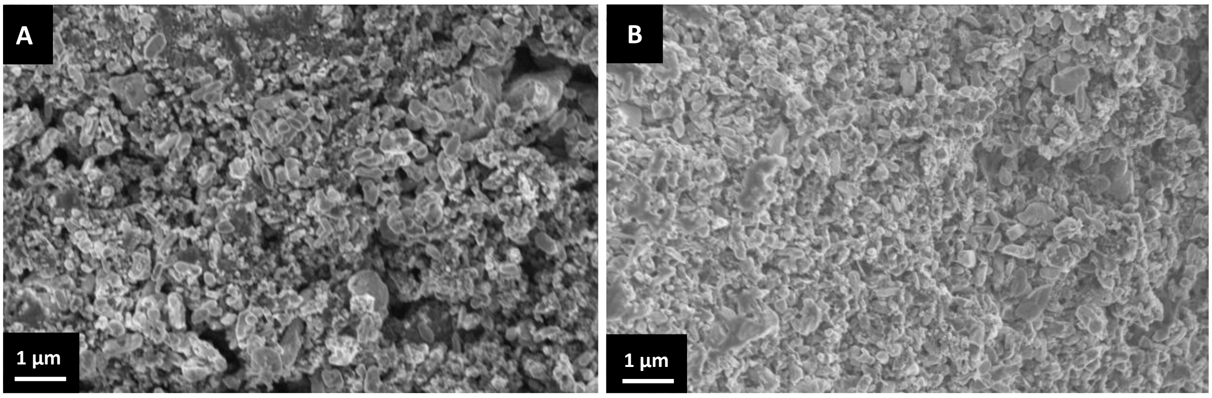

Figure S1: A) Scanning electron micrograph of the pristine LFP electrode and **B)** Scanning electron micrograph of the LFP electrode after 100 cycles in the process water solution.

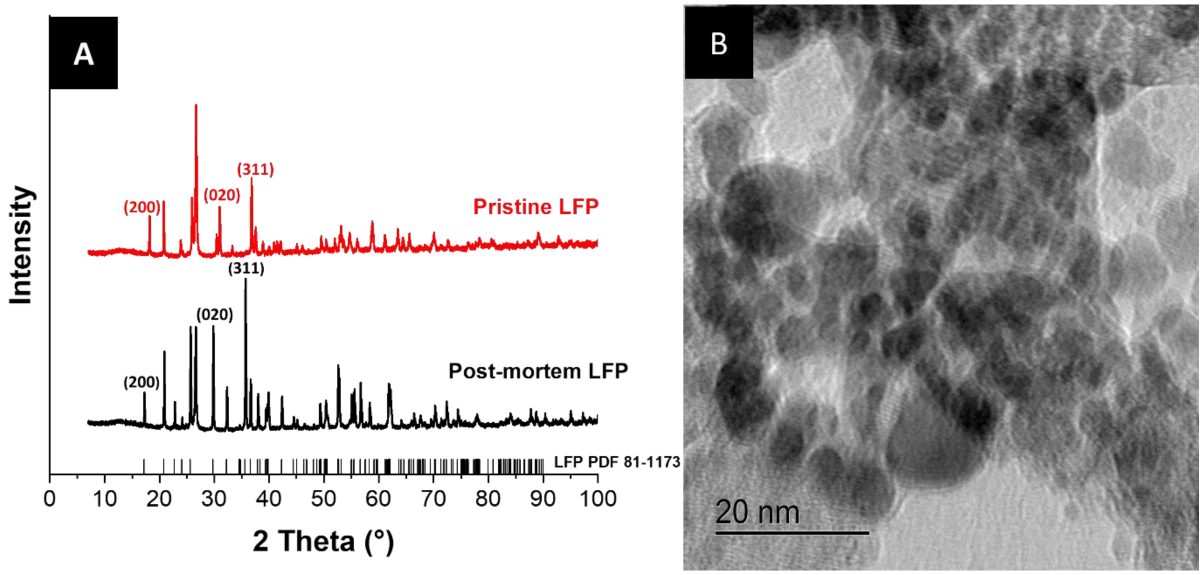

Figure S2: A) X-ray diffractogram of the pristine LFP electrode and of the post-mortem LFP electrode after being cycled in the process water solution for 100 cycles. **B)** Transmission electron micrograph of the pristine LFP.

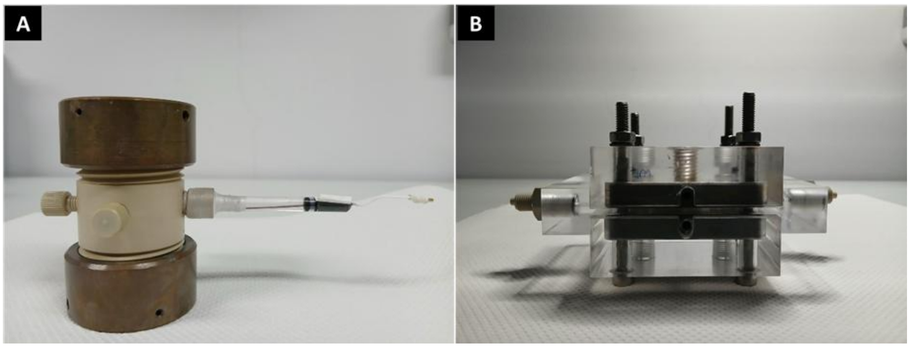

Figure S3: **A)** Photograph of static electrochemical cell. **B)** Photograph of the desalination cell.
